# Radiation Proctitis: Current Strategies in Management

**DOI:** 10.1155/2011/917941

**Published:** 2011-11-17

**Authors:** Nhue L. Do, Deborah Nagle, Vitaliy Y. Poylin

**Affiliations:** Division of Colon and Rectal Surgery, Beth Israel Deaconess Medical Center, Harvard Medical School Boston, 330 Brookline Avenue, Stoneman 9, Boston, MA 02215, USA

## Abstract

Radiation proctitis is a known complication following radiation therapy for pelvic malignancy. The majority of cases are treated nonsurgically, and an understanding of the available modalities is crucial in the management of these patients. In this paper, we focus on the current treatments of radiation proctitis.

## 1. Introduction

Radiation causes both apoptosis and cell death secondary to damage to DNA, proteins, and lipids and usually affects rapidly proliferating cells such as those found in cancer [1]. Today, radiation to the pelvis is an important constituent in the treatment of pelvic malignancies and is administered either as neoadjuvant or adjuvant therapy. Initially, radiation therapy (RT) was believed to provide no benefit for patients, especially patients with rectal cancer, due to the resistance of these cancers to radiation. Only later was it discovered that higher doses were required to overcome that resistance. However, higher doses of radiation have resulted in collateral damage to organs in or surrounding the field of radiation. Currently, the most frequent complication of radiation therapy, secondary to the utilization of higher doses, is proctitis after treatment for prostate cancer [2]. 

Radiation therapy can be administered via external beam radiation or radioactive implants termed brachytherapy. External beam radiation is typically administered by an external photon generator with a variety of sources including gamma rays, electron beams, and X-rays via a four-beam approach which results in significant exposure to surrounding organs [3]. Particular to prostate cancer treatment, certain regions of the rectum receive a dose similar to, if not equal to, the dose delivered to the prostate. Newer modalities of external beam radiation delivery attempt to deliver equivocal or higher doses but with decreased toxicity to surrounding areas; these modalities include three-dimensional conformal radiation therapy (3D-CRT) [4] and intensity-modulated radiation therapy (IMRT) [5]. 3D-CRT utilizes CT scanning to focus radiation fields with a constant dose rate [4], while IMRT utilizes computational methods for beam orientation and dose adjustments within the same beam field [5]. Both methods allow for the use of higher doses of radiation for target tissues with less exposure of normal tissue. Newer methods are currently being developed and tested, which utilize heavy particles such as protons and neutrons, to improve outcomes with reduced toxicity. These methods have the potential to deliver optimal doses of radiation to the target tissue with reduced damage to normal adjacent tissue; long-term outcomes are not clear, and these techniques currently come at significantly higher cost [3].

Brachytherapy has two methods of delivery that attempt to achieve the same goal of localized exposure of target tissue. The most common approach necessitates the implantation of radioactive pellets (typically iodine 125 or palladium 103) into the target tissue with a gradual release over time [6]. An alternative method involves the use of hollow catheters that can be progressively filled with increasing amounts of radioactive pellets over time. Brachytherapy reduces exposure damage to surrounding tissue based on careful positioning of the implanted pellets [6]. The rate of colorectal complications with brachytherapy is generally lower compared to external beam radiation. A study by Lesperance et al. show a decrease for both acute (6% versus 43%) and chronic (2% versus 21%) complications with brachytherapy when compared to external beam radiation [7, 8]. 

The incidence of radiation proctitis is not clear due to the lack of consensus on its definition and reporting methodologies. There is a general agreement that the incidence is likely related to the dose of radiation, area of exposure, method of delivery, and the use of cytoprotective agents. The doses generally delivered to the pelvis vary from 45 to 50 Gy for adjuvant or neoadjuvant treatment for prostate or anorectal malignancies; up to 90 Gy is considered the definitive therapy for gynecological malignancies [9, 10]. It is generally agreed that treatments <45 Gy cause very few side effects. Doses between 45 and 70 Gy, which is the dosage range for most treatments, cause more complications, but the complications tend to be of lesser intensity [9, 10]. Doses above 70 Gy cause significant and long standing injury to the surrounding area [9, 10]. The Radiation Therapy Oncology Group (RTOG) and the European Organization for Research and Treatment of Cancer (EORTC) individually attempted to classify the degree of radiation proctitis into a graded system of symptoms progressing from no symptoms to fatal complications (Table 1) [11, 12]. External beam radiation studies have seen incidence rates of radiation proctitis range from 2% to 39% [7–10] depending upon the severity/grade of proctitis, whereas IMRT studies have seen incidence rates from 1% to 9% [11–13] and particle radiation therapy in the range of 1% [14]. Although particle radiation therapy seems to have a lower incidence of proctitis, it is limited by cost and only available at specialized facilities. The incidence of proctitis in patients treated with brachytherapy alone is 8% to 13% and up to 21% when used in combination with other modalities [15]. 

Radiation proctitis is generally classified as acute or chronic, usually delineated by the timeframe of symptoms in relation to the treatment as well as the presenting symptoms and signs.

## 2. Acute Proctitis

Acute radiation proctitis is defined as an inflammatory process involving only the superficial mucosa [2] that occurs almost *immediately after the initiation of therapy or up to 3 months after the onset of therapy* [16]. Symptoms including diarrhea, nausea, cramps, tenesmus, urgency, mucus discharge, and minor bleeding will develop in up to 20% of patients necessitating an interruption in treatment [17]. Grossly, radiation proctitis is an inflammatory process of the rectal mucosa that appears edematous, beefy red, and may have ulceration or sloughing. Microscopically, there is a loss or distortion of the microvillus architecture with hyperemia, edema, and ulceration [2]. Colonoscopic biopsy of the inflammatory rectal mucosa is usually not recommended due to the increased risk of bleeding and fistula formation. Acute radiation proctitis does not increase the risk of chronic radiation proctitis [2] and is usually self-limiting with the discontinuation of the radiation therapy.

## 3. Chronic Proctitis

The time frame of chronic radiation proctitis can begin early, even during the acute phase of radiation proctitis, but symptoms may not become apparent until months to years later after the *cessation* of therapy (median 8–12 months after the completion of therapy) [2]. Symptoms of chronic proctitis may include those of acute radiation proctitis but may further include severe bleeding, strictures, perforation, fistula, and bowel obstruction. The pathological process is different from the acute phase and ultimately involves the compromise of blood supply to the rectal wall which results in full-thickness ischemia and fibrotic changes [2]. Grossly, the intestines are pale, noncompliant with telangiectasias, and may have strictures, ulcerations, fistulas, or heavy bleeding [18]. Microscopically, there is focal distortion and destruction of small arteries and arterioles with intimal fibrosis [18]. Chronic radiation proctitis has a significantly larger effect on the quality of life of individuals afflicted by this complication when compared to patients with acute proctitis [2].

The incidence of chronic radiation proctitis is estimated at 2%–20% [18]. Tissue biopsies are usually inconclusive with an inflammatory picture, and a diagnosis is made only after the exclusion of coexisting disease [2]. Since an alteration of blood supply, especially of small vessels, is a significant part of chronic radiation proctitis, conditions that further affect microvascular circulation such as diabetes and peripheral vascular disease may increase the risk of developing proctitis [9, 10]. Another mechanism that may have some contribution to the development of chronic radiation proctitis is oxidative stress; antioxidant agents are being utilized more recently to protect tissue damage in radiation injury [19]. Furthermore, it appears that patients with inflammatory bowel disease (IBD) may be at an increased risk of developing radiation proctitis as well as other complications from external beam irradiation [20]. A retrospective study by Willet, which included 28 patients with IBD, showed an overall incidence rate of complications of 46% at 32 months. The incident rate is higher than previous studies of patients without IBD and may be attributed partly to the inherent propensity of these patients to develop bowel complications as well as their immunosuppressed state from IBD steroid treatment [20]. There is some suggestion that limiting the exposure of already immunocompromised bowel, which is possible with the use of brachytherapy, may decrease the incidence of complications in patients with IBD [21]. 

While treatment and followup for HIV has improved to the point where it can now be considered a chronic condition, a number of HIV/AIDS associated malignancies have been rising. Some of these malignancies include cervical cancer, anal cancer, and lymphomas, all of which may require radiation therapy that can affect the rectum. There have been a number of reports suggesting higher complication rates in AIDS patients after radiation therapy. The etiology is unclear, but it has been suggested that the systemic glutathione deficiency seen in HIV patients leads to the depletion of radioprotective thiols and increased oxidative injury. For HIV positive patients with anal cancer and CD4 counts less than 200, Hoffman et al. [22] reports increased toxicity (bleeding, and mucositis) and decreased tolerance, thereby suggesting the need for lower treatment doses. Other studies suggest a similar toxicity profile [23] and recommend no change in the treatment dose. At this time, there are no reported conclusive data on radiation proctitis in patients with HIV/AIDS and prostate cancer. 

## 4. Diagnosis

Radiation proctitis should be suspected in any patient who has had pelvic radiation exposure and presents with the symptoms mentioned above. The majority are diagnosed after colonoscopy or sigmoidoscopy with features demonstrating pallor, friability, and telangiectasias. Other causes usually need to be excluded such as infection or inflammatory bowel disease. Fistulizing disease including rectovaginal, rectourethral, and rectovesicular fistulas could be late presenting symptoms of radiation proctitis but usually will be preceded by other symptoms.

## 5. Preventative Measures

Although modifications of radiation techniques and doses are continually being studied to decrease the incidence of radiation proctitis, trials investigating preventive methods have been disappointing to date. Small double-blind randomized control trials have shown no difference in the rates or severity of chronic radiation proctitis with the use of rectal (200 *μ*g) misoprostol and oral (3 gm b.i.d.) or rectal (3 gm daily) sucralfate [24–27]. Amifostine administered intravenously (340 mg/m^2^ daily) has shown some promise in small trials in preventing symptoms of acute proctitis as well as decreasing the severity of chronic proctitis symptoms. The follow-up period, however, was relatively short [28–30]. Overall, preventative measures have not made a significant contribution to decrease the incidence of radiation proctitis.

## 6. Treatments

### 6.1. Acute Proctitis

While acute proctitis is self-limiting, up to 20% of patients undergoing external beam radiation will require short interruptions in their treatments to improve symptoms. Supportive medical management is usually the only treatment required and includes hydration, antidiarrheals, and possibly steroid or 5-aminosalicylate enemas. Cessation of therapy is usually the definitive treatment and surgical interventions are rarely needed.

### 6.2. Chronic Proctitis

**Figure 1:**
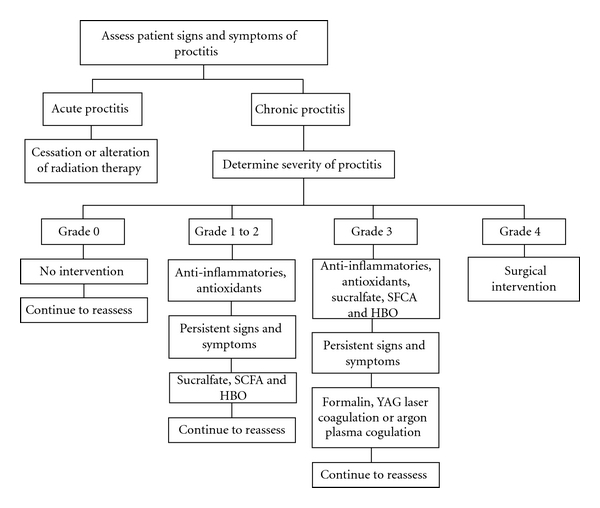


The management of chronic proctitis can be divided into noninvasive treatments (anti-inflammatory agents, sucralfate, short-chain fatty acids, hyperbaric, antioxidants) and invasive treatments (ablation and surgery). Although there is considerable variation in the management strategies for chronic proctitis, there is generally a strategy of using the least invasive interventions first with gradual progression as symptoms and signs worsen (Figure 1).

### 6.3. Noninvasive Treatments

Noninvasive therapy for chronic radiation proctitis begins with the use of oral, rectal, or gaseous agents. These agents consist of nonsteroidal anti-inflammatory drugs, sucralfate, short-chain fatty acids (SCFA), hyperbaric oxygen (HBO), and antioxidants.

#### 6.3.1. Anti-Inflammatory Agents

Anti-inflammatory agents such as sulfasalazine or 5-aminosalicylic acid (mesalamine) are usually first line treatments, but they have low efficacy even in combination with other agents such as steroids and antibiotics. The mechanism of action of both nonsteroidal anti-inflammatory agents listed above is thought to be through the inhibition of prostaglandin synthesis or via the lipoxygenase pathway of arachidonic acid metabolism [31]. Other suggested effects include the inhibition of folate-dependent enzymes and free radical scavenging activity [31]. Sulfasalazine is re-excreted in the bile after absorption via the small bowel and split into sulfapyridine and 5-aminosalicylic acid by colonic bacteria [32]. Studies have suggested that 5-aminosalicylic acid is the active metabolite of sulfasalazine [33]. A single randomized controlled trial by Kochhar et al. [34] comparing sulfasalazine (500 mg t.i.d) with rectal steroids (prednisolone 20 mg b.i.d.) to rectal sucralfate (2 gm b.i.d.) had a clinical improvement in 53% (8 of 15) of patients with oral sulfasalazine plus rectal steroids compared to a 94% (16 of 17) improvement with rectal sucralfate alone. Objective data from this study showed 47% (7 of 15) had improved endoscopic finding in the sulfasalazine plus rectal steroids group as compared to 71% (12 of 17) in the rectal sucralfate alone [34]. This study was limited by a very small sample size with a short followup of only 4 weeks. 

Other anti-inflammatory agents that are used in combination with sulfasalazine or 5-aminosalicylic acid include prednisone, betamethasone, hydrocortisone, and metronidazole. Steroids have multiple mechanisms of action that produce anti-inflammatory effects which extend from stabilization of lysosomes in neutrophils to prevent degranulation to upregulation of anti-inflammatory genes via binding to glucocorticoid receptors [35]. Metronidazole is a nitroimidazole whose complete mechanism of action has not been fully elucidated, but thought to be via the reduction of the nitro group in an anaerobic environment [36]. A study by Denton et al. [2] comparing rectal betamethasone (5 mg b.i.d.) to rectal hydrocortisone (90 mg b.i.d.) suggested that the later agent had improved control. The response, however, occurred only over the short follow-up period of 4 weeks. Another study by Cavcic et al. [37] compared the combination of oral metronidazole (400 mg daily × 3 days), mesalamine (1 gm daily × 3 days), and rectal betamethasone to oral mesalamine and rectal betamethasone. This study suggested that metronidazole may have synergistic effects with steroids in treating the symptoms of chronic proctitis. Both of the above-mentioned studies suffer from small sample sizes and short follow-up times which detract from any significant conclusions. 

 A newer chlorite-based anti-inflammatory agent which contains the active ingredient OXO-K993 and is administered intravenously, WF10, has recently been studied for the treatment of radiation-induced proctitis. It was initially developed as an adjunctive therapy for AIDS and was used in combination with antiretroviral and opportunistic infection prophylaxis regimens [2]. Its mechanism of action is believed to be through the modulation of the cell-mediated immune system by stimulating phagocytosis and downregulating antigen presentation to decrease immune activation [38]. It has been used for radiation-induced cystitis, proctitis, and mucositis only in Europe. Preliminary studies demonstrate that control of bleeding symptoms for years (median follow-up time 55 months) was obtained after two doses (0.5 mL/kg body weight per day); maintenance of effective results was achieved with repeat treatment 1-2 times per year [38]. This anti-inflammatory agent has not been approved by the FDA for use in the United States.

#### 6.3.2. Sucralfate

Persistent symptoms after treatment with anti-inflammatory agents such as 5-ASA for greater than five months necessitates treatment with sucralfate or pentosan polysulphate (PPS). Sucralfate is a highly sulphated polyanionic disaccharide (complex of sulfated sucrose and aluminum hydroxide) [39] that is thought to stimulate epithelial healing and the formation of a protective barrier [40]. A few reports, including a randomized placebo controlled study, showed benefits to both clinical and mucosal outcomes with rectal sucralfate (2 gm b.i.d.) [15]. Rectal sucralfate (3 gm) has also been shown to be superior to anti-inflammatory regimens in treating radiation proctitis [41] and can result in symptom relief in 75% of patients [21]. Pentosan polysulphate (PPS, a synthetic derivative of a glycosaminoglycan which is present in the surface of the bladder, vessels, and the gastrointestinal tract lining) is thought to reduce epithelial permeability and prevent adherence similar to sucralfate [42]. 

#### 6.3.3. SCFA

Short chain fatty acids (SCFA) are the main oxidative fuel of the colonic mucosa and also serve to stimulate colonic mucosal proliferation [43]. They are thought to be produced by the anaerobic bacteria of the colon from nonabsorbed carbohydrates. The most significant product of SCFA is butyric acid [44]. SCFA also exerts a vasodilatory effect on the arteriole walls to improve blood flow [45]. Radiation-induced injury results in ischemia and loss of microvillus architecture which may result in the impairment of SCFA absorption, thereby contributing to the changes seen with chronic radiation proctitis [3]. Supplementation with SFCA enemas may accelerate healing by improving the deficiency experienced by the colonocytes. Two randomized studies looked at butyrate enemas (40 mM butyrate) and found nonsignificant improvement in symptoms and signs contrary to case reports suggesting some benefit. Both studies, however, were severely underpowered [46–48]. Further studies are necessary to evaluate the possible benefits of SCFA for chronic proctitis.

#### 6.3.4. HBO

Hyperbaric oxygen therapy (HBO) stems from the pathological process of ischemia involving the compromise of blood flow to the rectal wall. The benefit of HBO is theoretically achieved through the decrease of tissue hypoxia, possibly through its angiogenic and antibacterial effects [49, 50]. Data on the use of HBO in chronic radiation proctitis is limited. A review by Bennett et al. [51] found an increased chance of improvement with hyperbaric oxygen treatment (RR 1.75, number needed to treat = 5). However, the degree of benefit, the cumulative effects, or duration of the response cannot be quantified because of the methodology and quality of the data. One randomized-controlled trial by Clarke compared patients treated with 90 minutes of 100% oxygen at 2 atmospheres with patients treated for 90 minutes with 21% oxygen at approximately 1 atmosphere. Results suggested some benefit with symptom improvement after 30 treatments [52]. Most studies demonstrate that HBO is expensive and not readily available in most areas except in highly specialized centers due to the requirement for multiple treatments and specialized equipment.

#### 6.3.5. Antioxidants

Oxidative stress is thought to be a major mechanism in the development of chronic radiation proctitis; agents with antioxidant properties have been used in an attempt to limit tissue damage in radiation injury. In a study by Kennedy et al., which only included 10 patients, the use of Vitamin E and C significantly decreased the rate of diarrhea and urgency [19]. The benefit of Vitamin A has been looked at by itself and in combination with other therapies. In a double-blind study by Ehrenpreis, the use of Vitamin A significantly reduced proctitis symptoms and the effects extended to patients in the placebo group after crossover [53]. Patel reported that the addition of Vitamin A to treatment with 8% formalin increased the success rate of formalin and shortened the time needed to achieve improvement [54].

### 6.4. Invasive Treatments

#### 6.4.1. Ablative Procedures

Ablative techniques are reserved for the treatment of symptoms refractory to medical management and include formalin, endoscopic coagulation, and argonplasma coagulation. These techniques can be associated with complications that include bleeding, perforation, fistulas, and stenosis.

#### 6.4.2. Formalin

In radiation proctitis, vascular telangiectasia and nonhealing mucosal ulceration, perhaps due to an underlying obliterative arteritis, may lead to severe recurrent hemorrhage. While there are no prospective studies of formalin treatment, formalin is considered to be a safe and effective way to treat radiation proctitis causing significant bleeding. Formalin scleroses and seals fragile neovasculature in radiation damaged tissues to prevent further bleeding through chemical cauterization [16, 55, 56]. Two methods of formalin application have been described since its first reported use in 1986 which are the rectal instillation of 4% formalin solution or direct topical application of a 10% formalin solution [55]. The topical application directly to the mucosa is thought to produce a more targeted local chemical cauterization; however, its success is entirely dependent on accurate localization. Topical formalin (10% formaldehyde solution) is generally applied through a rigid proctoscope, flexible endoscope, or by direct application with formalin soaked gauze [2, 57, 58] Contact with formalin for 2-3 minutes (until slight blanching of the mucosa is achieved) is allowed and believed to cause chemical cauterization. It is a procedure that can be performed in the office or procedure suite with reported success rates of 70%–80% with few patients requiring repeat application [55]. A few studies reported similar efficacy and a better complication profile with lower doses of formalin (down to 4% solution) [56], but only a few patients were examined. The perianal skin needs to be protected during the procedure to prevent stricture and skin damage. To prevent topical damage, flushing and irrigation through flexible endoscope has been proposed as a way of administration. Formalin application is generally safe, but bleeding, strictures, and fistulas have been reported. Direct injection of formalin allows for the more precise administration of treatment and potentially fewer strictures, but it is not feasible with large mucosal areas effected and could lead to fistula formation. Minor side effects were not frequently reported. The duration of the treatment effects cannot be assessed reliably from the data available but appear to last a minimum of 3 months. The absence of quality of life data means the impact of this treatment from the patient's perspective cannot be addressed. 

#### 6.4.3. Endoscopic Coagulation

A variety of endoscopic coagulation devices are effective for the control of radiation-induced bleeding through coagulation of focal bleeding telangiectasias. Most of the studies currently available are retrospective and show an improvement of symptoms and a decreased recurrence after treatment with YAG laser coagulation or argon plasma coagulation [2]. Both methods are based on the delivery of thermal coagulation and should be reserved for patients suffering from significant hemorrhagic proctitis [59]. Several treatment sessions are often required. 

Argon plasma coagulation uses high-frequency energy (monopolar diathermy) transmitted to the tissue through an ionized gas in a noncontact fashion [59]. Its ability to control bleeding throughout the gastrointestinal tract has been demonstrated, and it has very limited tissue penetration, making it attractive for treatment of superficial bleeding. Available studies are retrospective and have a small number of patients [60, 61]. However, all studies have shown higher hemoglobin and fewer symptoms in patients with hemorrhagic proctitis who failed medical therapy. Single sessions have been reported to significantly improve symptoms [62], but on average, two to three treatments are needed to achieve this result. Improvements persisted for a number of months after therapy was finished [2, 63]. Most of the complications reported were mild and included cramps, mucus discharge, and stricture [2]. However, significant complications including large ulcers, perforations, and rectourethral and rectovaginal fistulas have been reported. 

YAG lasers have the same theoretical benefit as argon plasma coagulation with a limited depth of penetration and the possibility for precise application. Evidence for their use is similar to those for argon plasma coagulation with only a few studies available, including those with fewer than 10 patients. [64, 65]. Based on studies currently available, YAG lasers may be useful in the treatment of radiation proctitis, but the data are not strong enough to support their wide use. For both argon plasma coagulation and YAG lasers, cost and availability may also present significant obstacles.

#### 6.4.4. Surgery

Although surgery is often necessary for a diagnosis of radiation proctitis and as an adjunct to some of the treatment options described above, it is considered a last resort for patients with radiation proctitis. Fewer than 10% [66] of patients ultimately require surgery. When required, it is directed at specific symptoms and complications of radiation proctitis such as intractable bleeding, perforation, strictures, and fistulas. Rarely, surgery has been used to treat uncontrollable pain. Gas and stool incontinence are common symptoms that often accompany these complications and should be considered when deciding on the need for surgical intervention. When surgery is being considered, the pathophysiology of radiation damage must be taken into account. Microvascular damage caused by radiation not only causes the symptoms of radiation proctitis, but also can significantly impair healing after any surgery. Fecal diversion with either a colostomy or ileostomy is a common reason patients are referred to surgeons. Diverting the stool stream decreases symptoms of pain, tenesmus, drainage, and infection but rarely eliminates them completely. An ostomy can also improve symptoms related to incontinence and stricture, but it has a limited effect on bleeding. At least one study [67] showed significant improvement in bleeding from a diverting loop colostomy but not complete resolution. Diversion will improve symptoms but without additional interventions, the improvements in symptoms are unlikely to persist after an ostomy reversal.

Rectourethral, rectovaginal and rectovesicular fistulas present with infections, pain, or incontinence symptoms and are one of the more common complications of proctitis. Surgical treatment options have traditionally included local excision and reconstruction such as an advancement flap. Due to poorly vascularized tissues and low healing rates, however, these interventions should not be used as the only treatment modalities. Additional options to improve success of fistula repairs include pedunculated flaps, such as a gracilis or martius flap, which are used to facilitate healing by introducing well-vascularized healthy tissue. Diversion of stool or the urinary stream with an ostomy or a suprapubic catheter should be considered in almost all cases where repair is attempted. In some patients, a complete diversion will improve symptoms and their quality of life to the point that they do not require any further intervention [66, 68–70] even though the underlying problem is not repaired. In cases of complicated fistulous disease, especially when accompanied by significant pain and incontinence, a proctectomy or pelvic exenteration with or without reconstruction is recommended. While this is a definitive treatment, it is accompanied by significant morbidity, including exceedingly high rates of anastomotic leaks in cases of reconstruction and high rates of perineal wound complications when reconstruction is not attempted [69, 70]. When considering reconstruction for these patients, a temporary diversion should be part of the initial operation. It is also important to discuss with patients that although these procedures may be a technical success, they often result in unacceptable long-term morbidity including complicated scarring, stricture, and incontinence. In cases of abdominal perineal resections, bringing a well-vascularized tissue to close the wound, such as rectus abdominus, gracilis or gluteal V-Y flap, will significantly decrease rates of postoperative wound complications. 

In cases of severe and intractable bleeding, surgical options are very limited, because a diversion will rarely control the bleeding completely. Diverting loop colostomy was described in one study as a successful way of controlling bleeding [67], but in many instances, proctectomy may be the only option available. When strictures are an issue, a diversion of the stool stream will often result in a significant improvement of symptoms. Other options include resection with reconstruction when the stricture is higher in the rectum or an advancement flap (mucosa or skin) when the rectal stricture is in the anus. In all cases, given the poor quality of the tissues, at least a temporary diversion should be considered. When surgical treatment is needed, most studies demonstrate poor outcomes with high complication rates (15%–80%), and a mortality of 3%–9% [66, 68–70]. 

## 7. Conclusion

Radiation proctitis is a relatively rare complication of radiation therapy. Rates of both acute and chronic proctitis have been decreasing with improved radiation therapy techniques that allow for the targeted delivery of higher doses of radiation. It is important to note that radiation proctitis is a result of radiation doses that are beyond the ability of the normal tissue to repair or recover from injury. There is recent evidence that the impairment of the rectum's ability to heal may also mean that other organs exposed to the same high radiation doses may be at increased risk of malignant transformation [71]. It has been suggested that patients exposed to higher doses of radiation may need to be more closely screened for other malignancies [71], but further studies need to be conducted before definitive recommendations can be made. Although there are no good preventive measures available at this point, most instances of proctitis are self-limited and respond to medical management. A combination of sucralfate, steroids, and pain control have been successful in most cases to improve symptoms. In more severe cases, especially with bleeding, chemical (formalin) or thermal (endoscopic coagulation) treatments have been successful. Surgery is rarely required to treat this condition, but when performed can lead to significant improvements. Surgery, however, also results in the increased risk of postsurgical complications. More studies are needed to prospectively look at both the prevention and treatment of radiation proctitis as well as patients with special consideration such as those with IBD and HIV/AIDS.

## Figures and Tables

**Table 1:** 

Grade	Symptoms or signs	Overall management
0	No symptoms	None
1	Occasional urgency and occasional pain; superficial ulceration <1 cm^2^, occult bleeding, and mild stricture	Outpatient management; no lifestyle adjustments
2	Intermittent urgency and intermittent pain; superficial ulceration >1 cm^2^, occasional bleeding, and moderate stricture	Outpatient management; some lifestyle adjustments
3	Persistent urgency and persistent pain; deep ulceration, persistent bleeding, severe stricture	Possible short hospital admission or minor surgical intervention; major lifestyle adjustments
4	Refractory urgency and uncontrollable pain; gross hemorrhage, perforation, fistula, complete obstruction	Long-term hospital admission or major surgical intervention
5	Sepsis, multiorgan failure, and death	Fatal complications
